# Wild African great apes as natural hosts of malaria parasites: current knowledge and research perspectives

**DOI:** 10.5194/pb-4-47-2017

**Published:** 2017-03-14

**Authors:** Hélène Marie De Nys, Therese Löhrich, Doris Wu, Sébastien Calvignac-Spencer, Fabian Hubertus Leendertz

**Affiliations:** 1 Project group Epidemiology of Highly Pathogenic Microorganisms, Robert Koch Institute, Berlin, Germany; a current address: UMI 233, Institut de Recherche pour le Développement (IRD), INSERM U1175, and University of Montpellier, Montpellier, France

## Abstract

Humans and African great apes (AGAs) are naturally infected with several species of closely related malaria parasites. The need to understand the origins of human malaria as well as the risk of zoonotic transmissions and emergence of new malaria strains in human populations has markedly encouraged research on great ape *Plasmodium* parasites. Progress in the use of non-invasive methods has rendered investigations into wild ape populations possible. Present knowledge is mainly focused on parasite diversity and phylogeny, with still large gaps to fill on malaria parasite ecology. Understanding what malaria infection means in terms of great ape health is also an important, but challenging avenue of research and has been subject to relatively few research efforts so far. This paper reviews current knowledge on African great ape malaria and identifies gaps and future research perspectives.

## Introduction

African great apes (AGAs) have been known to naturally host malaria parasites since the early 1920s, when researchers first described various *Plasmodium* species infecting chimpanzees (*Pan troglodytes*) and gorillas (*Gorilla* spp.), which are closely related to those infecting humans (Reichenow, 1920). To this day, it remains difficult to date the actual origins of the malaria parasites which infect humans (Sharp et al., 2013) as well as their emergence in human populations. However, the expansion of *P. falciparum* for instance, the most virulent *Plasmodium* parasite (White, 2003), is believed to have started in Africa at least thousands or even tens of thousands of years ago (Carter and Mendis, 2002; Rich et al., 2009; Tanabe et al., 2010; Volkman et al., 2001) and to have played a significant role in recent human evolution (Carter and Mendis, 2002). Malaria is currently geographically widespread and constitutes a heavy burden for human public health (WHO, 2015). Malaria in great apes, our closest evolutionary relatives, has thus increasingly been subject to research as this leads to a better understanding of human malaria and the significance and implications of malaria parasite infection for great ape population health.

Research on AGA malaria parasites was initially limited to the study of captive and laboratory individuals (Bray, 1960; Contacos et al., 1970; Garnham et al., 1956; Rodhain and Dellaert, 1943; Rodhain, 1936, 1940; Schwetz, 1934). However, the development of molecular diagnostic tools and, later on, the ability to use non-invasive samples, i.e. the discovery that malaria parasite genetic material can be found in faecal samples of primates (Prugnolle et al., 2010), opened up opportunities for further research in wild ape populations. This allowed for more accurate identifications, the discovery of additional *Plasmodium* species in chimpanzees and gorillas, and the study of their phylogenetic relationships (Kaiser et al., 2010; Liu et al., 2010, 2014). Most of the current knowledge concerns malaria parasite diversity, host specificity and evolutionary history. There is still relatively little understanding of the epidemiology, ecology and biology of great ape malaria, but with increasing efforts being made in recent years to investigate these aspects. Here, we review up-to-date knowledge about the natural history of malaria parasite infection in wild AGAs, highlight the gaps in the knowledge and conclude with some perspectives for further investigation.

## Parasite diversity, distribution and prevalence in African great apes

Malaria parasites naturally infecting AGAs were initially described in the 1920s by Reichenow (Reichenow, 1920) and then studied further and experimented on by various researchers such as Blacklock and Adler (Adler, 1923; Blacklock and Adler, 1922), Schwetz (Schwetz, 1934), Rodhain (Rodhain and Dellaert, 1943; Rodhain, 1936, 1940), Brumpt (Brumpt, 1939), Garnham (Garnham et al., 1956), Bray (Bray, 1958a, 1960) and Contacos (Contacos et al., 1970). Based on morphological features, three distinct *Plasmodium* species, considered similar to the human parasites, were identified in West and Central Africa: *P. falciparum*-like and *P. ovale*- or *vivax*-like parasites in chimpanzees and gorillas and *P. malariae*-like parasites in chimpanzees (Reichenow, 1920). These three species were subsequently named *P. reichenowi* (*P. falciparum*-like parasites) (Blacklock and Adler, 1922), *P. schwetzi* (*P. ovale* or *vivax*-like parasites) (Brumpt, 1939) and *P. rodhaini* (*P. malariae*-like parasites) (Brumpt, 1939). *P. falciparum* and *P. reichenowi*, considered relatively distinct from other known *Plasmodium* species, were grouped separately under the *Laverania* subgenus (Bray, 1958b).

**Figure:**
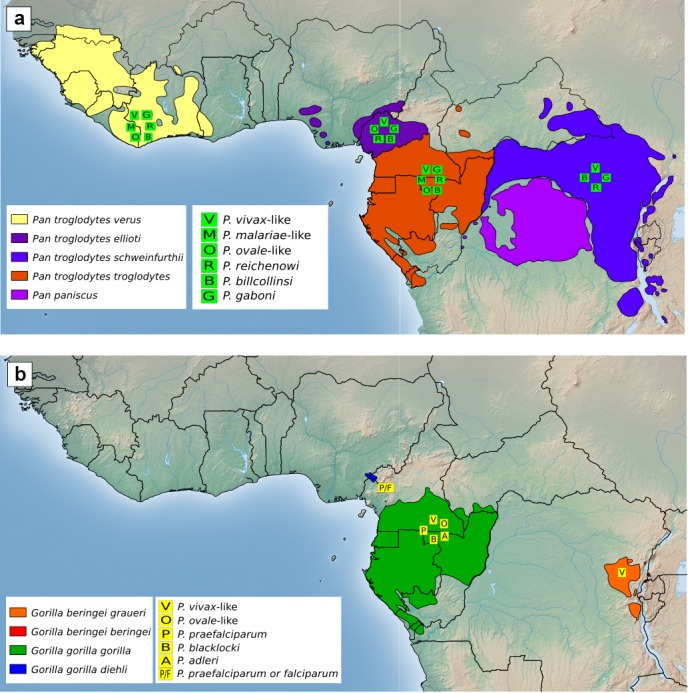
Distribution of *Plasmodium* species detected in wild chimpanzees and gorillas. *Plasmodium* species symbols placed on the geographical range of chimpanzee subspecies **(a)** and gorilla species and subspecies **(b)** indicate that the assigned *Plasmodium* species was, to this date, detected in at least one sample of the relevant great ape species and subspecies. Positions of the *Plasmodium* symbols do not represent specific field site locations.

From 2009 onwards, with the increased use of molecular diagnostic tools (deoxyribonucleic acid, DNA, amplification and genome sequencing) and, later on, the development of non-invasive methods to detect malaria infection through the amplification of parasite DNA from faecal samples, large-scale studies on malaria parasites infecting wild great apes flourished. These studies led to the description of a higher diversity of parasites than previously thought and gave clearer insight into prevalence rates and the possible origins and evolutionary history of malaria infection in humans. To date, at least six *Plasmodium* species have been shown to be endemic in wild-living chimpanzees: three *Laverania* species – *P. reichenowi* (Boundenga et al., 2015; Kaiser et al., 2010; Liu et al., 2010, 2016; Prugnolle et al., 2010; Rich et al., 2009) and two newly discovered *Laverania* species, *P. gaboni* (Boundenga et al., 2015; Kaiser et al., 2010; Liu et al., 2010, 2016; Prugnolle et al., 2010; Rich et al., 2009) and *P. billcollinsi* (Boundenga et al., 2015; Krief et al., 2010; Liu et al., 2010, 2016); and three non-*Laverania* species – *P. ovale*- (Kaiser et al., 2010; Liu et al., 2010), *vivax*- (Kaiser et al., 2010; Liu et al., 2010, 2014) and *malariae*-like (Kaiser et al., 2010; Liu et al., 2010) parasites (putative nomenclature was proposed by Rayner et al., 2011, and used in the present review). All four chimpanzee subspecies are naturally infected with all known chimpanzee *Laverania* parasites and with at least one of the non-*Laverania* parasites (Fig. 1a). Western lowland gorillas (*Gorilla gorilla gorilla*) also naturally harbour three parasite species belonging to the *Laverania* subgenus, i.e. *P. praefalciparum*, *P. adleri* and *P. blacklocki* (Liu et al., 2010, 2016; Prugnolle et al., 2010), and two non-*Laverania* species, i.e. *P. vivax*- (Liu et al., 2014) and *ovale*-like parasites (Mapua et al., 2015) (Fig. 1b). A *P. praefalciparum*, or *falciparum*-like, sequence (an undistinguishable sequence) was also detected in Cross River gorillas (*Gorilla gorilla diehli*) (Prugnolle et al., 2010), and *P. vivax*-like parasites were detected in eastern lowland gorillas (*G. b. graueri*) (Liu et al., 2014). Despite significant search efforts (861 samples tested by Liu et al., 2010, 2014), natural infections of wild-living bonobos (*Pan paniscus*) have not been reported so far (Fig. 1).

Before large-scale screenings on wild great ape populations were carried out, the natural prevalence of malaria parasites in some chimpanzee and gorilla populations was suspected to be high given the frequent detection in sporadic blood or tissue samples (Kaiser et al., 2010; Rich et al., 2009) as well as in faecal samples (Krief et al., 2010; Prugnolle et al., 2010) from wild-living chimpanzees (all four subspecies) and western gorillas. This was confirmed when prevalence levels of *Plasmodium* spp. infection in wild apes were estimated using detection rates from larger sets of faecal samples. Parasites were clearly found to be endemic in the four chimpanzee subspecies and in lowland gorillas (*G. g. gorilla* and *G. b. graueri*), with, according to different studies, estimated prevalence levels ranging from 32 % to 48 % for *P. t. ellioti*, *P. t. troglodytes*, *P. t. schweinfurthii* and *G. g. gorilla*, in which faecal detection rates were corrected for the sensitivity of the diagnostic test, sample degradation and redundant sampling (Liu et al., 2010); 15.42 % for *P. t. troglodytes* and 21.28 % for *G. g. gorilla* faecal detection rates (Boundenga et al., 2015); and 33 % for *P. t. verus*, in which the point prevalence rate was based on the proportion of positive individuals tested by faecal detection (De Nys et al., 2013, 2014). The vast majority of parasites harboured by AGAs belong to the *Laverania* clade with non-*Laverania* strains rarely found (Boundenga et al., 2015; Kaiser et al., 2010; Liu et al., 2010; De Nys et al., 2013, 2014). The specific prevalence rates of *P. vivax*-like parasites in chimpanzees (*P. t. troglodytes* and *P. t. schweinfurthii*) and lowland gorillas (*G. g. gorilla* and *G. b. graueri*) seem to range from 4 to 8 %, close to the infection rates in endemic human populations (Liu et al., 2014). Co-infection with two or more *Plasmodium* parasite species is prevalent (Krief et al., 2010; Liu et al., 2010), e.g. present in 55 % of positive chimpanzee samples tested by Liu et al., (2010). Bear in mind that these prevalence rates are likely to represent underestimates due to the lower sensitivity of faecal testing compared to blood testing (Liu et al., 2010, 2014). In some chimpanzee and gorilla communities, no malaria parasites were detected so far (Boundenga et al., 2015; Liu et al., 2010, 2014), which could be the result of the relatively low sensitivity of faecal detection combined with the small sample size (Liu et al., 2014). Additionally, low prevalence or absence of malaria parasites in certain communities could also be explained by environmental factors – for example, factors that lead to a scarcity of efficient vectors or demographic factors such as variations in host density.

Until the discovery of other *Laverania* parasites, *P. reichenowi* was known as the closest relative of *P. falciparum* (Escalante and Ayala, 1994; Escalante et al., 1995). Consequently, it was the first great ape *Plasmodium* species to be molecularly characterised. A number of phylogenetic analyses have led to various hypotheses on the origin of the human *P. falciparum*: (1) one is the co-speciation hypothesis, in which the two *Plasmodium* species diverged from a common ancestor at the same time as the chimpanzee and human lineages diverged, about 6 to 10 million years ago (Escalante and Ayala, 1994; Escalante et al., 1995; Hayakawa et al., 2008; Hughes and Verra, 2010); and (2) another is the hypothesis of a more recent chimpanzee origin through a single host transfer of *P. reichenowi* from chimpanzee to human. The latter theory was supported by the low genetic polymorphism of *P. falciparum* which was fully included into the broader genetic diversity of malaria parasite lineages, which the authors collectively considered as forming the species *P. reichenowi* (Prugnolle et al., 2010; Rich et al., 2009). However, recent broad-scale investigations based on the detection of malaria parasite DNA (mitochondrial, nuclear and apicoplast sequences) in faecal samples of wild AGA populations resulted in the genetic characterisation of a wide array of other *Plasmodium* species. This showed that the entire diversity of human-derived *P. falciparum* sequences is encompassed by the gorilla *P. praefalciparum* clade (Liu et al., 2010; Sundararaman et al., 2013) and led to the substantial indication that *P. falciparum* originated from a relatively recent zoonotic transmission of *P. praefalciparum* from gorillas to humans (Liu et al., 2010).

As far as non-*Laverania* parasites are concerned, many questions remain on their evolutionary origins and the phylogenetic relationships between the parasites hosted by non-human primates (NHPs) and those hosted by humans. Phylogenetic analyses initially suggested that *P. vivax* originated in Asia from an ancestral macaque *Plasmodium* species (Escalante et al., 2005; Mu et al., 2005). *P. simium*, a New World monkey parasite, has also been designated as a potential ancestor of *P. vivax* given their high genetic similarity, which is suggestive of a recent host transfer between New World monkeys and humans, although the direction of this cross-species transmission remains uncertain (Escalante et al., 2005; Tazi and Ayala, 2011). Finally, the near fixation of the Duffy-negative phenotype in Sub-Saharan human populations, which confers resistance against *P. vivax*, supports the hypothesis of an African origin of this parasite with positive selection of the Duffy negativity through long-term exposure (Carter, 2003). The *P. vivax* strains found in captive AGAs are genetically distinct from those circulating in humans (Prugnolle et al., 2013). They include the monophyletic lineage formed by the human *P. vivax* parasites in their diversity, but they lack host specificity amongst the AGAs they infect, i.e. *P. t. troglodytes*, *P. t. ellioti*, *P. t. schweinfurthii*, *G. g. gorilla* and *G. b. graueri* (Liu et al., 2014). This supports the idea that the human *P. vivax* developed from within a *Plasmodium* species which also infected African apes and that all present human *P. vivax* parasites originated from an ancestor which spread out of Africa (Liu et al., 2014). For *P. malariae*- and *ovale*-like parasites, larger-scale investigations of infections in wild NHPs together with phylogenetic analyses are still needed to clarify the evolutionary relationship of these parasites with human strains.

## Transmission of AGA malaria parasites

### Vectors

It is well established that mosquitoes are responsible for the transmission of malaria between humans. These malaria vectors belong to the *Anopheles* genus (*An.*), of which approximately 40 species (out of 430) are thought to be competent for malaria parasite transmission (CDC, 2012). They have been studied extensively and mapped in detail (Sinka et al., 2010). The ecology of mosquito species, including habitat and host preferences, varies and determines their importance as a vector in given habitats (Sinka et al., 2010). For instance, *An. gambiae* is considered as one of the most efficient vectors in open landscapes in Sub-Saharan Africa (Coetzee, 2004; Sinka et al., 2010). Additionally, *An. moucheti*, also highly anthropophilic, is entirely restricted to forested habitats and considered a significant vector of human malaria in this type of environment (Sinka et al., 2010).

Until very recently, not a single vector responsible for AGA malaria parasite transmission had been identified. Early experiments had shown the susceptibility of some known human malaria vector species to *P. reichenowi* (Blacklock and Adler, 1922; Collins et al., 1986), with a notable exception being *An. gambiae*. Bray (1958a) had managed to infect *An. gambiae* with the formerly called *P. schwetzi*; however, the low levels of sporozoites in the salivary glands had suggested that these were not the natural vectors. Another experiment succeeded in infecting humans with *P. schwetzi* via *Anopheles balabacensis* bites (Contacos et al., 1970).

It is mainly since the discovery of the gorilla origin of *P. falciparum* that the question of AGA malaria vectors has been increasingly raised due to the concern for future zoonotic transfers of malaria parasites from NHPs to humans and thus the importance of identifying species that can act as bridges (Verhulst et al., 2012). At the present time, several studies have thus specifically searched for mosquito species responsible for the transmission of malaria parasites between chimpanzees or gorillas in their natural habitat. The first study tested female *Anopheles* (N=100) captured at chimpanzee (*P. t. schweinfurthii*) natural nesting and feeding sites in Uganda, but it did not detect any *Plasmodium* parasites (Krief et al., 2012). The failure of detection might, however, have been linked to the small number of specimens tested. A second study, which screened a larger number of female *Anopheles* (N=1070) captured in areas hosting AGA populations in Gabon, found DNA from *P. praefalciparum* in one *An. moucheti* and from *P. vivax*-like parasites in one *An. moucheti* and one *An. vinckei* (Paupy et al., 2013). Finally, the analysis of 2415 female *Anopheles* captured in the same areas in Gabon led to the discovery of both chimpanzee and gorilla *Laverania* and non-*Laverania* parasites in *An. vinckei*, *An. moucheti* and *An. marshallii* (Makanga et al., 2016), with the highest prevalence rate in *An. vinckei* (N=35). The detection of sporozoites in their salivary glands confirmed their role in natural transmission. These mosquito species appeared to be non-host-specific (they were shown to feed on humans and to carry malaria parasites from rodents, bats and antelopes) and were thus unlikely to be responsible for the host specificity of *Laverania* parasites.

It is important to note that the prevalence rates of different mosquito species might differ between geographical sites, suggesting a variability of vectors depending on the investigated sites. For example, mosquito trappings performed in and around the forest of the Taï National Park (TNP) in Côte d'Ivoire to study the distribution of mosquito genera in habitats varying from human settlements to a primary forest only identified one single *Anopheles* in the primary forest (at a camp site), whereas they were abundant in the neighbouring villages (Junglen et al., 2009). Mosquitoes of the *Uranotaenia* genus, however, were dominant in the primary forest but rarely found in other areas. This suggests that the malaria vectors responsible for the transmission of malaria parasites in the resident chimpanzee population differ from those transmitting parasites in humans and in other AGA populations. Obviously, other mosquito genera and other dipterans should not be excluded as possible vectors. Biting midges (genus *Culicoides*), for example, transmit *Hepatocystis* spp., which are bat and monkey malaria parasites (Blanquart and Gascuel, 2011), and are known to be abundant in the TNP (personal observation).

### Interspecies transmission

Since the discovery of AGA malaria parasites, the marked similarities they share with those infecting humans have raised the question of whether the exchange of these parasites between AGAs and humans could occur, experimentally and/or naturally.

Early experiments involving captive chimpanzees and humans (volunteers and/or neurological patients for former therapeutic purposes) demonstrated that, for some strains, the infection of humans with chimpanzee parasites, or vice versa, is possible. This was the case for the chimpanzee-borne *P. rodhaini* (probably a *P. malariae*-like strain) (Bray, 1963; Rodhain and Dellaert, 1943; Rodhain, 1940), the human-borne *P. malariae* (Bray, 1960, 1963; Garnham et al., 1956), the chimpanzee-borne *P. schwetzi* (probably *P. ovale*- or *vivax*-like strains) (Bray, 1963; Coatney, 1968; Contacos et al., 1970; Rodhain and Dellaert, 1955) and the human-borne *P. vivax* (Bray, 1963; Garnham et al., 1956; Rodhain, 1939). In contrast, attempts to transfer the *Laverania* species *P. reichenowi* from chimpanzees to humans failed (Blacklock and Adler, 1922; Rodhain, 1939). The marked host specificity of *P. reichenowi* might be due to the human loss of the common primate Sia (sialic acid) *N*-glycolylneuraminic acid, which is the preferred target of *P. reichenowi* for erythrocyte binding, as opposed to *P. falciparum*, which prefers the precursor of the *N*-glycolylneuraminic acid, the Sia *N*-acetylneuraminic acid (Martin et al., 2005). In addition, the transfer of *P. falciparum* to chimpanzees only seemed possible in splenectomised individuals, or limited to the liver or to a very low parasitaemia in intact individuals (Bray, 1963; Taylor et al., 1985).

For certain human malaria parasites, natural (non-experimental) transmission to AGAs seems to occur. De facto, early experimental attempts at infecting chimpanzees with *P. falciparum* gave poor results, but captive bonobos (Krief et al., 2010) and chimpanzees (Duval et al., 2010; Ngoubangoye et al., 2016) living in areas of high human *P. falciparum* endemicity have been found to be infected with these parasites. These infections were probably acquired from humans (Rayner et al., 2011). There is, however, no information on the viability of such infections and thus the potential for vector-based transmission between apes. Similarly, *P. malariae*, which has never been detected in wild bonobos so far, was found in captive individuals (Krief et al., 2010), and a *P. ovale* strain, identical to the human *P. ovale* variant type, was found in a captive chimpanzee (Duval et al., 2009).

As for natural zoonotic transmission of AGA parasites to humans, it appears from the phylogenetic relationships between known malaria parasites that, historically, ape *Plasmodium* (*P. praefalciparum*) have managed to naturally cross the species barrier at least once (Liu et al., 2010). This, together with some of the old experimental results reported above, instinctively leads to the assumption that natural zoonotic transfers of AGA malaria parasites might still occur, leading to new host switches or just to regular transmission events in the same manner that a *P. knowlesi* infection in humans originates from regular zoonotic transfer events from Southeast Asian macaques (Lee et al., 2011; Sharp et al., 2013). Interestingly, the AGA malaria parasite vectors found by Makanga et al. (2016) are mosquito species that also seem to feed on other mammalian species, including humans. They could thus, in theory, act as vectors for interspecies transmissions. Studies led in Cameroon (Sundararaman et al., 2013) and Gabon (Délicat-Loembet et al., 2015) searched for evidence of zoonotic transfers of ape *Laverania* species by testing large numbers of human blood samples (N=1402 and N=4281) from people living in the vicinity of chimpanzee and gorilla populations with high *Laverania* prevalence rates. No ape parasites (*Laverania* or non-*Laverania*) were detected in these samples, implying that zoonotic transfers might be rare events.

There is, however, current evidence that cross-species transmission from wild apes to humans of the non-*Laverania* species *P. vivax* is ongoing. Since wild chimpanzees in West and Central Africa were confirmed hosts of *P. vivax*-like strains, it was hypothesised that wild apes could act as a natural reservoir (Rayner et al., 2011), thereby explaining the occasional infection of travellers despite the low prevalence of *P. vivax* in the human populations of these regions (Broderick et al., 2015; Culleton et al., 2008; Gautret et al., 2001; Mühlberger et al., 2004; Skarbinski et al., 2006). Moreover, a *P. vivax*-like parasite isolated from a traveller coming back from a Central African forest was recently shown to cluster with the AGA isolates outside of the human lineage, confirming the occurrence of cross-species transmissions (Liu et al., 2014; Prugnolle et al., 2013). This is supported by the fact that *P. vivax*-like parasite DNA was found in an anthropophilic mosquito species, *Anopheles moucheti* (Paupy et al., 2013). Given the close resemblance to their human counterparts and past experimental findings, it is likely that, similarly to *P. vivax*, zoonotic transfers of *P. ovale*- and *malariae*-like parasites from chimpanzees also take place. It would be useful to characterise more of these parasites in order to search for evidence of such transmissions (Sundararaman et al., 2013).

Transmission of host-specific parasites between different AGA genera has only, so far, been observed in captivity, where great ape species cohabit closely, e.g. transmission of *Laverania* parasites from chimpanzees to gorillas and vice versa (Ngoubangoye et al., 2016). Cross-species transmission between AGAs and other NHPs sharing their habitat is probably exceptional. So far, the only published finding showing such potential is that of *P. praefalciparum* detected in a single captive greater spot-nosed monkey, *Cercopithecus nictitans* (Prugnolle et al., 2011). Yet, during a subsequent study which tested blood from a significant number of wild spot-nosed monkeys (N=292), only *Hepatocystis* spp. and one *Plasmodium* sp. previously described in birds and lizards were found (Ayouba et al., 2012). *P. vivax* has also specifically been searched for in blood samples (N=998) from 16 Old World monkey species; however, with the exception of *Hepatocystis* spp., no malaria parasites were found (Liu et al., 2014). Similar results were obtained after screening specimens from six monkey species from Uganda (N=102) (Thurber et al., 2013) and from three monkey species in the TNP, Côte d'Ivoire (N=38) (S. Calvignac-Spencer, personal communication, 2015). *Hepatocystis* represents one of the numerous genera of malaria parasites, just like the genus *Plasmodium*, which seems to be paraphyletic to *Hepatocystis* (Martinsen et al., 2008). *Hepatocystis* parasites comply with a broad pattern of host-specificity, with distinct lineages that specialise on monkeys and bats (Ayouba et al., 2012; Schaer et al., 2013). So far, there is no published evidence of transmission of NHP malaria parasites such as *Hepatocystis* spp. to AGAs. Moreover, amongst 30 chimpanzee carcasses from the TNP (Côte d'Ivoire) that were tested for malaria parasites, no *Hepatocystis* spp. were found (S. Calvignac-Spencer, personal communication, 2015). Interestingly, however, a liver sample from one of these chimpanzees tested positive for a saurian *Plasmodium* parasite.

## Extrinsic and intrinsic determinants of *Plasmodium* infection

Extrinsic factors, such as environmental factors (habitat type and climate), group size and host density as well as possibly behavioural factors, such as sleeping site choice, most likely influence the exposure to malaria parasites and thus the frequency of infections in specific AGA communities. However, only very few studies so far have investigated their effects, which remain largely unknown.

The influence of monthly rainfall on the probability of *Plasmodium* detection in chimpanzee and gorilla faecal samples was tested in a single study so far, but no significant effect was found (Boundenga et al., 2015). Yet, it was shown that rainfall can affect *Plasmodium* parasite prevalence in the vectors of AGA malaria parasites, with increased prevalence and thus exposure during the rainy season (Makanga et al., 2016). Prevalence in mosquitoes was also shown to vary with canopy height, with higher rates at mid-height compared to ground and canopy level.

There is very limited information on the effects of host group size and sleeping site, mainly drawn from studies on New World primates. In contrast to the encounter-dilution effect, which predicts a decreased risk of exposure to an infected vector with increasing group size (Krebs et al., 2014; Mooring and Hart, 1992), malaria prevalence in New World primates was found to increase with average sleeping group size, suggesting that malaria might be a significant cost associated with larger group sizes (Davies et al., 1991; Nunn and Eckhard, 2005). However, data on primate malaria and group size are still too scarce to draw any firm conclusions. Sleeping site selection in these primates also seems to play a role in malaria infection rates by reducing the risk of attack by the vector (Nunn and Eckhard, 2005). In chimpanzees, two studies looked at malaria and sleeping sites indirectly by capturing mosquitoes at natural nest sites. While one study failed to detect any patterns of association between the choice of nest site and mosquito densities (Koops et al., 2012), the second study found patterns compatible with mosquito avoidance (Krief et al., 2012). Chimpanzees seemed to choose nest sites in drier and higher places compared to their feeding sites, and these sites were indeed characterised by lower densities of *Anopheles* mosquitoes. Mosquitoes were also less abundant in sites with more nests (Krief et al., 2012). Moreover, an experimental study showed that tree species preferred by chimpanzees for nest building might be selected due to their repulsive properties against flying arthropods (Samson et al., 2013).

As for intrinsic determinants of malaria infection in AGAs, based on data from human malaria, one would expect factors such as age, pregnancy status, co-infections, immune status and genetics to influence susceptibility to infection as well as levels of parasitaemia and the expression of clinical malaria.

As a matter of fact, patterns strikingly similar to those found in human populations from malaria endemic areas have been observed in wild AGAs. Faecal detection rates of *Plasmodium* infection were found to be higher in younger individuals of *P. t. verus* (De Nys et al., 2013) and *G. g. gorilla* (Mapua et al., 2015) as well as in pregnant females of *P. t. verus* (De Nys et al., 2014). This probably reflects changes in the frequency of new infections and/or in parasite densities induced by variation in susceptibility to these parasites due to age and pregnancy status. These fluctuations in susceptibility might be the result of immune protection acquired in adults through constant exposure to the parasites (Doolan et al., 2009) and, during pregnancy, a combination of immunological and hormonal changes, together with the ability of *P. falciparum*-infected erythrocytes to sequester in the placenta (Rogerson et al., 2007). However, variations in the exposure to the vectors and, thus, to malaria parasites might also occur and play a role in these observed patterns. It was, for instance, demonstrated in humans that pregnant women are more attractive to *Anopheles* mosquitoes compared to non-pregnant women, probably for physiological reasons, e.g. increased exhaled breath and abdominal temperature (Ansell et al., 2002; Lindsay et al., 2000). Additionally, possible behavioural changes, such as nesting habits, that could lead to increased exposure to the vectors should be considered.

In addition, it would be judicious to keep in mind that in a given host, malaria parasites are part of a parasite community, whereas we use the term parasite in its broad ecological sense, designating micro- and macroparasites (Faure, 2014). This implies that, just like in an ecosystem, multiple and complex interactions between these parasites and with their host (i.e. their environment) take place; this can also partly dictate the presence of parasite species or their densities in a specific host, as opposed to a single host–parasite relationship (Pedersen and Fenton, 2007; Rynkiewicz et al., 2015), and perhaps explain (at least partially) the patterns of malaria parasite detection observed in AGAs. As an illustration, Telfer et al. (2010) actually demonstrated, for the first time in a natural population of field vole (*Microtus agrestis*), that co-infection with several microparasites explains more of the variation in the risk of infection compared to other factors such as host condition, e.g. age, and exposure risk, e.g. season (Telfer et al., 2010). Co-infection interactions can take place via competition for shared resources, e.g. nutrients, physical site of infection, or the immune system of the host, e.g. cross-reactivity or immunosuppressant pathogens such as HIV (Graham, 2008; Pedersen and Fenton, 2007; Rynkiewicz et al., 2015). Resource-mediated parasite interactions would be likely to occur between malaria parasites and other haemoparasite species which target the same cells, like erythrocytes. For instance, *Babesia microti* infection in *Microtus agrestis* negatively affects host susceptibility to *Bartonella* spp. infection and vice versa (Telfer et al., 2010). Likewise, a negative association between *Plasmodium* spp. and *Babesia* spp. has been recently observed in wild Malagasy primates (*Propithecus verreauxi*), where high *Babesia* spp. infection levels in younger individuals also seem to confer protection against *Plasmodium* spp. infections, possibly via shared resource competition (Springer et al., 2015). Inhibition of *Plasmodium cynomolgi* infection by *Babesia microti* was also previously described in experimental rhesus macaques (van Duivenvoorde et al., 2010; Voorberg-vd Wel et al., 2008). In humans, there is evidence of a pathological interaction between the immunosuppressant HIV virus and *Plasmodium*, with HIV-seropositive people being at increased risk of *Plasmodium* infection and the development of clinical malaria (Patnaik et al., 2005; Whitworth et al., 2000). Malaria parasites and various helminth species have also been shown to interact in humans, with either an antagonistic or beneficial relationship, possibly mediated by cross-reactive immune responses (Faure, 2014). In order to fully understand the patterns of malaria infection in AGAs, it would thus be necessary to also explore and take into account co-infections with parasites that are likely to interact.

Genetic factors may additionally play a role in conferring a certain degree of resistance to infection by malaria parasites or clinical disease. Genetic regulation of the immune response by certain alleles encoding the major histocompatibility complexes I and II, several interleukins, the CD40 ligand, the nitric oxide synthase, and interferon α and γ receptors has been associated with protection against severe malaria in humans (Hill et al., 1991; Kwiatkowski, 2005). Mutations leading to variants of erythrocyte characteristics also provide protection against parasite invasion – for example, the Duffy negativity for *P. vivax*; glycophorin A, B and C deficiency for *P. falciparum*; and haemoglobin E (β-globin gene mutation) – or confer protection against clinical malaria, such as haemoglobin S (sickle haemoglobin) and C (both β-globin gene mutations), glucose-6-phosphate dehydrogenase (G6PD) deficiency and $\alpha^{+}$-thalassemia (defective production of α globin) (Kwiatkowski, 2005; Williams, 2006). To date, there is very little information available for AGAs. The diversity of the G6PD and β-globin genes has been examined in chimpanzees, but so far no mutations appear to have been selected for malarial resistance (MacFie et al., 2009; Verrelli et al., 2006). Moreover, Liu et al. (2014) sequenced samples from 134 AGAs but did not find any Duffy-negative phenotypes.

## Pathogenicity of malaria parasites in African great apes

None of the published studies have focused on the consequences of malaria infection on wild great apes' health so far, and the information collected from experimental or captive conditions is rather limited. Observations made during early experiments on the clinical course of malaria in captive chimpanzees, i.e. mainly the lack of noticeable illness behaviour during various infections with human and possibly chimpanzee strains, led Rodhain (1936, 1940) to believe that, in natural conditions, chimpanzees do not suffer significantly from malaria and that they can be subject to asymptomatic chronic infections lasting several months. On one occasion, he reported a double inoculation with *P. falciparum* and *P. vivax* in a young individual, which had induced a febrile state with loss of appetite but only mildly (Rodhain, 1936). The only indications on the duration of infections also come from early experimental studies. The infections seemed to be able to last for at least several months, with periods as long as 5 months recorded for *P. reichenowi* and *P. schwetzi* (Garnham et al., 1956) and over 1 year for *P. malariae* (Rodhain, 1940).

Reports from sanctuaries or other facilities hosting captive individuals are rare and variable. In Japan, two captive chimpanzees were found to be chronically infected with *P. malariae*, without showing any evidence of illness for 30 years (Hayakawa et al., 2009). *P. falciparum* infection of captive bonobos in the Democratic Republic of the Congo were also not associated with apparent clinical signs or increased body temperature (Krief et al., 2010). Moreover, a young chimpanzee newly transferred to a sanctuary after 6 years of captivity in an urban setting developed severe anaemia and hyperthermia concomitant with a high *P. reichenowi* parasitaemia (Herbert et al., 2015). The latter case would suggest that infection with certain malaria parasites can, under certain circumstances, be detrimental for chimpanzees' health. However, extrapolating these observations to the wild is difficult as stress related to captivity could also influence the effect of such infections. Moreover, whether individuals were initially naïve to the *Plasmodium* strains they were diagnosed with (whether human or chimpanzee derived) is generally undetermined (strains novel to a chimpanzee's immune system would be more likely to cause illness). One publication also reported the death of a 1-year-old captive chimpanzee infected with *P. reichenowi*; however, given the extremely poor and stressful conditions this individual was kept in, whether malaria effectively played a significant role in the fatal outcome is questionable (Tarello, 2005).

In contrast, pathogenicity has never been demonstrated in AGAs living in natural conditions so far. The general impression given by the above-mentioned elements, as well as by the absence or rarity of reported malaria-like clinical signs or death associated with infection in individuals observed on a sometimes daily basis (wild habituated or captive individuals) (e.g. Kaiser et al., 2010), is that wild-living chimpanzees are mostly undisturbed by malaria infections, or that at least the clinical impact and related discomfort are generally mild enough to be invisible to observers' eyes when not specifically searched for. Also, as pointed out by Rayner et al. (2011), the very high prevalence rates of malaria parasite infections observed in numerous wild chimpanzee and gorilla populations indicate that severe outcomes are probably rather rare.

As young and pregnant chimpanzees seem to be more susceptible to malaria infection, it is possible that they are also more prone to suffer from the biological consequences of infection and to develop clinical malaria, as seen in humans (Doolan et al., 2009). Healthy and non-pregnant adults probably benefit from a certain degree of acquired clinical immunity, but it would be reasonable to think that malaria infection would start having some level of detrimental effect when the host's ability to control parasite density is diminished. Higher levels of blood parasitaemia are likely to at least induce higher degrees of anaemia (Kotepui et al., 2015; Oni and Oguntibeju, 2006), which in turn could cause morbidity or even mortality. Anaemia is actually recognised as one of the major causes of morbidity and mortality related to malaria in young children and pregnant women (Ekvall et al., 2001). Moreover, in humans, malaria during pregnancy can have deleterious consequences for the pregnancy's outcome and the infant's health (Desai et al., 2007; Guyatt and Snow, 2001). Deleterious effects on pregnancy outcome and/or infant health would mean a general impact on population reproductive success and fitness, thereby shaping population dynamics.

Further investigation of the relationship between malaria parasites and chimpanzees in natural conditions is important to understand the significance of malaria infection for wild great ape populations' health, and it can serve as a model to understand the host–parasite relationship in human populations, where malaria is believed to have acted as an important driver of recent evolution (Carter and Mendis, 2002; Sabbatani et al., 2010). Wild habituated AGA communities are ideal targets to pursue investigations on illness related to malaria infection. In a more indirect way, studying the AGA malaria parasites' genes, which might play a role in pathogenesis, can also provide valuable information. For example, the very diverse *var* genes, found only in *Laverania* parasites, are unique to *P. falciparum* in humans and play an important role in its pathogenesis by coding for proteins which mediate the cytoadherence of infected erythrocytes (Smith et al., 2013). Analysis of *var* and *var*-like gene sequences from human and ape *Laverania* species showed that many characteristics of these genes are conserved across the different *Laverania* species and that these evolved well before the emergence of all the *Laverania* species (Larremore et al., 2015). In addition, the *var2csa* genes, known to be associated with the adherence of *P. falciparum*-infected erythrocytes to the placenta and placental malaria in humans, have also been identified in *P. reichenowi* and *P. gaboni* (Pacheco et al., 2013; Trimnell et al., 2006). However, the question of whether the expression of *var* genes is also associated with pathogenicity in AGAs remains open. Some significant differences have also been found between *var* genes from *P. gaboni* and *var* genes from *P. falciparum* and *P. reichenowi*, suggesting potential disparities in their biology.

## Conclusions and perspectives

It is apparent that, to date, most of the knowledge on wild AGA malaria is concentrated around parasite diversity, distribution and phylogenetic relationships with malaria parasites infecting other species, humans in particular. Indeed, the majority of research studies ultimately aim at identifying potential animal reservoirs with ongoing zoonotic transmission as well as assessing the risk of emergence of new malaria strains in human populations (by, inter alia, exploring the origins of the current human malaria parasites). Many gaps, however, still need to be filled in order to achieve these objectives – e.g. further exploration of vectors, of phylogenetic relationships of ape-derived *P. malariae* and *P. ovale* strains with human strains, and of zoonotic infections in humans living close to AGA communities.

The epidemiological determinants and the effects of malaria infection in wild AGAs have been left largely unexplored. This also leaves many questions that need to be addressed in order to further understand the basic epidemiology and biology of malaria parasites in AGAs. What are the influences of other known intrinsic and extrinsic factors affecting human malaria on infection, parasite density or, perhaps, disease in AGAs? Are there visible patterns of infection in specific populations? How frequently and for how long are individuals infected? What are the pathological consequences of malaria infection? Does infection have a significant impact on great ape health? Could the impact of malaria be sufficiently subtle to be misleading? Is this just like SIV (simian immunodeficiency virus) infection in chimpanzees, which was believed for many years to be rather trivial for their health (non-pathogenic), but whose actual deleterious impact on their health only became apparent recently when examined at the level of an entire community (Keele et al., 2009; Rudicell et al., 2010)? Does malaria play a role in sociality, demographics, population dynamics and general fitness? Are there any indications of selective pressures exerted by malaria on AGA evolution or development of resistance? These are just a fraction of the multiple questions that still need to be addressed. Using faecal samples has proven suitable and efficient, not only to address broad-scale questions such as those on the geographic distribution and genetic diversity of great ape malaria parasites, but also to address small-scale epidemiological questions which might, in comparison, require a higher (finer-scale) resolution and thus more sensitive screening (De Nys et al., 2013, 2014). As non-invasive methods to study pathogen infections and monitor health parameters (e.g. body temperature, inflammation markers and hormone levels) are now available, attempts to answer some of these questions are actually conceivable. In the meantime, further exploration, improvement and testing of non-invasive methods need to be pursued in order to enhance sensitivity, facilitate the interpretation of results and broaden research possibilities.

## Data Availability

This is a review article; therefore, no data were obtained.
